# Stricture of the Colon

**Published:** 1857-05

**Authors:** G. R. Henry

**Affiliations:** Burlington, Iowa


ART. II.—*Stricture of the Colon. Amussat's Operation.* By G. R. HENRY, M.D., of Burlington, Iowa.

ON Wednesday, the 3d of December, 1856, I was called to see Mr. J. M. Swan, æt. 45, of nervous bilious temperament, who told me there had been no action on his bowels since the Saturday previous, and but little for a week or ten days prior to that time.

During the past two years I had been called to see him several times, on account of constipation, which I had hitherto been able to remove by the ordinary cathartics, and which I had attributed to the sedentary life he was leading. I supposed this attack would yield as the former ones had done, although he told me he had taken several doses of senna tea without effect. I gave him a full dose of calomel and Dover's Powder, directing it to be followed the next morning with the black draught. I called about noon the next day; found there had been no action upon the bowels, but great commotion in the small intestine. I directed enemas, which brought away nothing but a few scybalæ. Up to this time

Mr. Swan had been eating heartily, his appetite being unimpaired; I directed a reduction in the amount, and that the food should be of the most laxative nature.

December 5th, I gave croton oil in large doses, producing slight nausea, but no discharge. I introduced a large gum catheter ten inches into the bowel, and threw up about three pints of salts and senna; this remained some time, but brought nothing away with it. After this he could not bear an enema of more than half a pint, and this but for a few minutes. I went through the usual routine of purgatives, calomel, aloes, rhubarb, elaterium, scammony, gamboge, croton oil, &c., in different combinations; injections of all kinds, both warm and cold, were resorted to; galvanism was tried, but without avail. Thinking that there might possibly be spasmodic stricture, I gave antim. tart. for two days in full doses, which produced but little nausea after the first few doses, and accomplished nothing.

On the night of the 8th, I gave a grain of sulph. morphine, under which my patient slept well, and in the morning seemed much refreshed. During the whole of his sickness, his urinary secretion was copious, but high-colored. On the morning of the 9th I became convinced that there was an *organic* obstruction—and from the absence of vomiting, the distension of the colon, and the evident action of the upper bowel under the use of purgatives, I was satisfied that the trouble was in the lower part of the colon.

On the morning of the 10th, my patient was much worse; haggard in appearance; pulse 140; abdomen tympanitic and very much swollen, and suffering so from pain and distension, that he could not sleep at all, even under the free use of opium. I felt convinced that unless relief was afforded, he would die within twenty-four hours, and so told him, mentioning the operation and its chances. At 12, M. he consented, and at 2 P.M. I operated upon the descending colon, as directed by Amussat. I was assisted by Drs. Rauch, Webb, McCann, and Scarf; Drs. J. F. Henry, and Ransom also being present. The operation was attended with but little loss of blood; a large amount of fecal matter was discharged from the wound, and the patient expressed much relief. I dressed the wound by the interrupted suture, attaching the edges of the bowel to the integument; a free discharge was kept up all the evening, and his pulse fell to 120; the next morning he was entirely comfortable, pulse 110. December 13th, patient said he felt "first-rate;" about 2 P. M. I was sent for in haste, and found him almost in *articulo mortis* from hemorrhage of a venous nature; I opened the wound to the bottom, and found the bleeding proceeded from a small portion of the cellular tissue surrounding the kidney; the organ itself was not injured, but there appeared to be a congeries of little vessels in the cellular tissue, from which the blood poured as from a sponge. I

applied ice, gave stimulants, and there was no return of the bleeding. I could not close the wound, and the discharge of fecal matter kept it in a condition unfavorable for healing.

Two days elapsed before he rallied from the hemorrhage, he then seemed to improve; his pulse fell to 80, and he felt that he was going to recover.

This state of things continued until Friday, the 19th, when he became much distressed by the attempted passage of scybalæ through the wound; some of these I cut to pieces and extracted, but as I would remove one, another would pass down, causing much pain, and producing a diarrhœa, with constant desire to have an operation both by the wound and the natural passage; through the latter, a little fluid bilious matter was discharged, being the first which had passed the anus for three weeks. This pain and diarrhœa continued, in spite of all I could do, through the night of the 19th, and on the morning of the 20th, being the tenth day after the operation, between eight and nine o'clock, he expired, apparently from exhaustion.

AUTOPSY, three hours after death. Present, Drs. J. F. Henry, Rauch, Webb, and McCann. The bowels healthy to the immediate vicinity of the wound, but containing large scybalous masses, from the size of a marble to that of a goose-egg, which were so hard as to be cut with difficulty, and almost all of them having, as a nucleus, portions of apple-parings, although the patient had not eaten an apple for two months before his death. The number of these scybalous masses was large, there being more than a gallon of them, and many had passed the wound at the time of the operation and daily afterwards; but there were some too large to pass, and seemed to be insoluble in the secretions of the bowel, or in the water which was injected through the wound to soften them; the largest mass was in the cœcum.

The inflammation about the wound was much less than I supposed it would be, extending but a few inches from the incision.

Fourteen inches below the opening, and ten from the anus, just below the sigmoid flexure, a stricture, embracing all the coats of the bowel, was found, which effected almost a complete closure of the tube, leaving a mere fissure, through which the flat end of a small scalpel handle could with difficulty be passed. A small ulcer appeared on the superior side of the stricture, and extended down into it for a couple of lines; the stricture was six lines long, and the part of the gut embraced in it, was perfectly callous, cutting like gristle; indeed, so firm and unyielding, that it is not likely that any mode of treatment would have proved effectual for its dilatation; so that had the patient survived the operation which was performed, he would have continued to suffer under the infliction of that disgusting alternative, an artificial anus.

I found in this case, by following the directions of Amussat, that the wound extended farther back than was necessary, leaving the bowel in the anterior portion of the cut, and injuring the lumbar muscles more than there is any necessity for, and causing the operator to endanger the kidney. If I had to operate again, I should commence the incision one inch farther forward than he directs, and shorten it by that much posteriorly.

Of course the operator should be governed by the case before him; but the modification suggested would, in my opinion, render the operation a much simpler one, in not requiring so deep a wound, and not subjecting the patient to the risk of hemorrhage, while it would place the outlet of the bowel in a much better position for dressing than when, as now, it is at the most elevated portion of the incision.

